# Functional Analysis and Proteomics Profiling of Extracellular Vesicles From Swine Plasma Infected by African Swine Fever Virus

**DOI:** 10.3389/fcimb.2022.809135

**Published:** 2022-02-10

**Authors:** Guowei Xu, Xijuan Shi, Huanan Liu, Chaochao Shen, Bo Yang, Ting Zhang, Xuehui Chen, Dengshuai Zhao, Jinke Yang, Yu Hao, Huimei Cui, Xingguo Yuan, Xiangtao Liu, Keshan Zhang, Haixue Zheng

**Affiliations:** State Key Laboratory of Veterinary Etiological Biology, National Foot-and-Mouth Disease Reference Laboratory, Lanzhou Veterinary Research Institute, Chinese Academy of Agricultural Science, Lanzhou, China

**Keywords:** ASFV, extracellular plasma vesicles, swine, function, proteomics profile

## Abstract

African swine fever (ASF) has brought excellent barriers to swine production in China and the world. Studies have shown that extracellular vesicles mediate the RNA and protein spread of pathogenic microorganisms and RNA and proteins. After infection by pathogenic microorganisms causes significant differences in the proteins contained within extracellular vesicles. Based on the above studies, the extracellular vesicles were extracted from ASF virus (ASFV)-infected swine plasma. And qPCR, western blot, and confocal experiment were carried out. The research shows that extracted extracellular vesicles significantly promote the replication of ASFV in susceptible and non-susceptible cells Proteomics analysis of the extracellular vesicle proteins revealed that ASFV infection could cause significant differences in the protein profile. This study demonstrates that extracellular vesicles play a critical role in ASFV replication and transmission and cause significant differences in the protein profile encapsulated in extracellular vesicles.

## Introduction

African swine fever (ASF) is a severe hemorrhagic, febrile, hemorrhagic, and highly contagious infectious disease in pigs caused by the ASF virus (ASFV) and result in a fatality rate as high as 100% (Malogolovkin and Kolbasov, 2019; Sanchez et al., 2019; Wade et al., 2019). ASFV is the only member of the Asfivirus genus and the Asfaviridae family (Ge et al., 2018; Olesen et al., 2018), and have a giant double-stranded DNA virus, with an average diameter of 200 nm.

Extracellular vesicles are intracellular vesicles secreted by most model cells with a size of 40~150 nm (Kalluri and LeBleu, 2020). The formation of extracellular vesicles requires the transformation of early endosomes into late endosomes. Multivesicular bodies (MVBs) sprout inward to form smaller intraluminal vesicles (ILV), and ILVs released from the cell after fusing with the cell membrane. The formation of extracellular vesicles is believed to involve endosome sorting complexes (Colombo et al., 2014). There are significant differences in the biological functions of exosomes from different cell sources, which are often related to the stimulation of secreting cells by external factors that cause changes of bioactive substances significantly such as RNA, proteins, and liposomes in the cell (Colombo et al., 2014; Maas et al., 2017). Extracellular vesicles have several characteristics like certain viruses, including biogenesis, cellular uptake, and the transfer of functional RNA, mRNA, and proteins between cells (Meckes and Raab-Traub, 2011; Yao et al., 2018; Neerukonda et al., 2019; Zhang et al., 2019; Mao et al., 2020; Xu et al., 2020a; Luong and Olson, 2021; Xu et al., 2021; Zhang et al., 2021). The differences between extracellular vesicles and some viruses include self-replication after infecting new cells, temporarily regulated viral expression, and the complexity of viral entry (Alenquer and Amorim, 2015). Extracellular vesicles released by B lymphocytes stimulate specific CD4+ T cell clones *in vitro*, which indicates that they serve as carriers for MHC II peptide complexes between immune system cells (Raposo et al., 1996; Vincent-Schneider, 2002; Montecalvo et al., 2008).

The protein components of extracellular vesicles have a vital role in the biological function of extracellular vesicles. Because of the diversity of protein molecules in extracellular vesicles, their study from the perspective of markers or disease mechanisms will become an important area of investigation. Interestingly, after infection by pathogenic microorganisms, the proteins, genetic material, and even whole virions are also contained in the extracellular vesicles secreted by the infected cells. Studies have shown that extracellular vesicles mediate the spread of various pathogenic microorganisms, such as the Porcine reproductive and respiratory syndrome virus(PRRSV), Foot-and-mouth disease virus (FMDV), Seneca valley virus (SVV), and Hepatitis C virus (HCV) (Correia et al., 2013; Takamatsu et al., 2013; Franzoni et al., 2018). Meanwhile, extracellular vesicles transmit those biologically active substances between cells, and mediate signal transduction, resulting in biological function changes. Recent studies have demonstrated that extracellular vesicles are essential in virus pathogenesis and immunity (Harendra et al., 2015).

Upon infecting the host, ASFV causes changes in biologically active substances, including protein, RNA, miRNA, and these changes are often important factors that alter cellular processes (Correia et al., 2013; Takamatsu et al., 2013; Alejo et al., 2018; Franzoni et al., 2018; Kessler et al., 2018; Molini et al., 2020; Barroso-Arévalo et al., 2021). The role of porcine plasma extracellular vesicles in the host antiviral immune response is unknown, especially during ASFV infection. Whether extracellular vesicles mediate the spread of ASFV and its genome and proteins are still unknown. Also, the mechanism through which ASFV infection results in changes in biologically active substances contained in extracellular vesicles has not been elucidated.

To clarify the role of pig plasma extracellular vesicles in the process of ASFV infection, we extracted and identified extracellular vesicles from the plasma of ASFV-infected and healthy pigs. The extracted extracellular vesicles were incubated into ASFV non-susceptible cells, where we found that ASFV-EVs (ASFV-extracellular vesicles) promoted ASFV replication in susceptible and non-susceptible cells. We further conducted a proteomic analysis of ASFV-EVs and MOCK-EVs (MOCK-extracellular vesicles) and identified many differentially expressed proteins in ASFV-EVs, including significant differences in ASFV proteins. Gene Ontology (GO) annotation and the KEGG pathway analysis determined potential functions for these proteins.

## Materials and Methods

### Cells and Viruses

HEK-293T and MA-104 cells were obtained from the American Type Culture Collection. PK-15 cells was obtained from the China Cell Resource Bank. The cells were maintained in DMEM supplemented with 2 mM L-glutamine, 100 U/ml gentamicin, nonessential amino acids, and 10% fetal bovine serum. As previously described, the ASFV isolates, CN/GS/2018, were propagated on PAM(Porcine alveolar macrophages) cells (García-Belmonte et al., 2019).

### Animal Experiments

Commercially bred swine (80~90 lb) was inoculated with ASFV CN/GS/2018(ten HAD_50_ for each pig). Plasma was collected from 3 pigs with viremic on the 6th day after ASFV inoculated (Li et al., 2021). The plasma of three ASFV-negative pigs was collected at the same time.

### Extracellular Vesicles Isolation and Purification

To further separate and purify the collected supernatant, we performed differential centrifugation as follows. All centrifugation steps were carried out in a 4°C environment. The supernatant was initially collected by centrifuging at 500 × g for 5 minutes to remove larger fragments and cells. The cell debris was further removed by centrifugation at 2,000 × g for 10 min. The resulting supernatant was centrifuged at 12000 × g for 45 minutes to remove the cells. The large vesicles were collected and filtered through a 0.22 um filter. Finally, the collected supernatant was centrifuged at 120,000 g for 2 h using an ultracentrifuge (Thermo Scientific Sorvall WX100). The resulting precipitate was resuspended in 500 μL of PBS.

### Nanoparticle Tracking Analysis (NTA)

The mean size and size distribution profile of ASFV-EVs were analyzed as previously described (Luong and Olson, 2021). Briefly, the samples were diluted at a ratio of 1:1000 in PBS containing 0.05% Tween-20 in a total volume of 1.0 mL. Measurements were performed in triplicate using standard settings (refractive index = 1.331, viscosity = 0.89, and temperature = 25°C). Data analysis was performed using NTA 3.2 software (Malvern Panalytical Ltd., Malvern, Worcestershire, UK), and the samples were evaluated using the Nanosight NS300 (Malvern Panalytical Ltd., Malvern, Worcestershire, UK).

### Transmission Electron Microscopy (TEM)

Direct morphological observation of the extracellular vesicles is crucial for extracellular vesicles identification. Therefore, we analyzed the extracted extracellular vesicles using TEM (Hitachi H-7000FA, Tokyo, Japan). After observation, we first extracted the extracellular vesicles using a TEM 200 copper mesh (EMS 80100-Cu US), then staining with phosphoric acid for 2 min. After drying under an incandescent lamp, electron microscopy was used to observe the extracted extracellular vesicles using a voltage of 80 kV.

### Western Blot Analysis

For western blot (WB) analysis, the purified extracellular vesicles were lysed with radio-immunoprecipitation assay buffer (Santa Cruz Biotechnology, Dallas, TX, USA), and the cleared lysate was collected by centrifugation for protein separation on 12% sodium dodecyl sulfate-polyacrylamide (SDS-PAGE) gels. After electrophoresis, the separated proteins were transferred to 0.45 μm polyvinylidene difluoride (PVDF) membranes (Millipore, USA). The membranes were blocked for 1 h with Tris-buffered saline containing Tween 20 (TBST) with 5% non-fat milk. The blots were then incubated with primary antibody at 4°C overnight. The primary antibodies used included mouse monoclonal anti-CD63 (Abcam, Cambridge, UK), anti-ASFV p30 (Prepared by our laboratory), rabbit monoclonal anti-CD9 (Abcam, Cam bridge, UK), anti-APOA1(Abcam, Cam bridge, UK), rabbit polyclonal anti-ASFV p72 (prepared by our laboratory), SERPINC1 (Abcam, Cam bridge, UK). After washing three times with TBST, the membranes were incubated with horseradish peroxidase (HRP)-labeled secondary antibody (Proteintech, Chicago, IL, USA) for 2 h at room temperature. Finally, the proteins were visualized with Clarity enhanced chemiluminescence (ECL) WB substrate (Bio-Rad Laboratories, Hercules, CA, USA).

### Analysis and Quantification of ASFV DNA

For qPCR detection of ASFV DNA, total DNA from ASFV-EVs samples was isolated with the E.Z.N.A. total DNA kit I (Omega Bio-Tek) to quantitate DNA copies of ASFV in ASFV-EVs. After DNA extraction, the cartridge was processed for qPCR. Briefly, the target for amplification of the ASFV genome was a conserved p72 gene region using the following primers: 5′-ctgctcatggtatcaatcttatcga-3′ and 5′-gataccacaagatc(ag)gccgt-3′, and a TaqMan probe (5′-[6-carboxy-fluorescein]-ccacgggaggaataccaacccagtg-3′-[6-carboxy-tetramethyl-rhodamine], Applied Biosystems) was designed from an alignment of 54 available ASFV sequences of the 3′-end of p72. Analysis was performed using MxPro software, and the quantitative PCR procedure included the following thermocycling steps: denaturation (95°C), annealing (58°C), and elongation (72°C). The amount of ASFV genome was calculated using the standard curve and expressed as genome copies per milliliter.

### Extracellular Vesicles Mediate ASFV Replication in ASFV-Susceptible and Non-Susceptible Cells

The extracted extracellular vesicles were inoculated into susceptible cells (BMDM) and non-susceptible cells (HEK-293T, PK-15) 8 h after inoculation with ASFVThe extracted ASFV-EVs (25ng) were inoculated into susceptible cells (BMDM) and non-susceptible cells (HEK-293T, PK-15) 8 h, meanwhile PBS was used as control, and then inoculated with ASFV(MOI:0.01).After 24 h of inoculation with ASFV, the cells and culture supernatant were collected, and DNA was extracted to detect the viral load of ASFV in the samples by qPCR and WB analysis. MOCK-EVs and ASFV-EVs were pre-stained with DiL and then inoculated into susceptible cells (BMDM) and non-susceptible cells (PK-15, HEK-293T) for 8 h. The nuclei were stained with DAPI(4’,6-diamidino-2-phenylindole), and fluorescence was measured by confocal laser microscopy.

### Differentially Expressed Protein Analysis

The Proteome discoverer 2.4 software was used to perform qualitative and quantitative calculations on the proteomics data for TMT markers. Analysis of the quantitative results of the pairwise comparison groups revealed differential expression using a threshold of greater than 1.2-fold (up and down) and a *P*-value <0.05. Proteins meeting this screening criterion were considered significantly differentially expressed. The properties of the genes and gene products in organisms may be categorized as Biological Process, Molecular Function, and Cellular Component. The GO annotation for the target protein set is derived from the biological processes involved. The proteins are classified into the terms molecular function and cellular components. The significant enrichment analysis of GO annotation using Fisher’s exact test was done to evaluate the significance level of the GO terms. KEGG pathway enrichment analysis is like GO enrichment analysis. The KEGG pathway is used as a unit, and all qualitative proteins are used as the background. Fisher’s Exact Test was used to analyze and calculate the significance level of protein enrichment for each pathway to determine whether it significantly affected metabolism and signal transduction.

### Biosafety Statement and Facility

All experiments with live ASFVs were conducted within the enhanced biosafety level 3 facilities at the Lanzhou Veterinary Research Institute of the Chinese Academy of Agricultural Sciences and approved by the Ministry of Agriculture and Rural Affairs and the China National Accreditation Service for Conformity Assessment.

### Ethics Statement

According to the Animal Ethics Procedures and Guidelines of the People’s Republic of China, all animals were handled i strictly according to good animal practices. The study was approved by the Animal Ethics Committee of Lanzhou Veterinary Research Institute of the Chinese Academy of Agricultural Sciences.

## Results

### Isolation and Identification of ASFV-EVs

**Figure 1:**
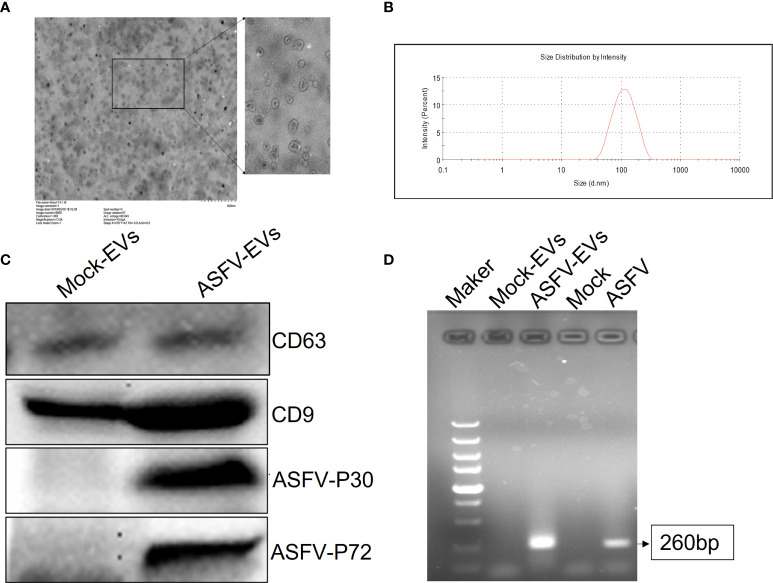
Plasma exocytosis maker protein and ASFV protein contained in extracellular vesicles were identified. **(A)** The extracted extracellular vesicles were stained with phosphotungstic acid and observed with a transmission electron microscope. **(B)** The purified plasma extracellular vesicles samples were diluted 1:1000 in PBS containing 0.05% Tween-20 to a total volume of 1.0 mL. The test was repeated 3 times with standard settings (refractive index = 1.331, viscosity = 0.89, temperature = 25°C). Each sample was analyzed 5 times, and the average value was taken. **(C)** Extracellular vesicles CD63 and CD9, ASFV P30 and P72 proteins were identified in extracellular vesicles by WB analysis. **(D)** PCR identification results of ASFV p72 geneMOCK-EVs represent extracellular vesicles extracted from ASFV-negative pig plasma, ASFV-EVs represent extracellular vesicles extracted from ASFV-positive pig plasma, Mock represent PBS negative control, and ASFV represent ASFV-positive cell culture supernatant.

At present, the identification of extracellular vesicles is mainly carried out by observing the morphological size and detecting related maker proteins. We first found that the extracted extracellular vesicles were especially cup-shaped, and there were no other multivesicular structures by TEM (**Figure 1A**). The size of the extracted extracellular vesicles was measured by nanometer particle size, and the results showed that the particle size of ASFV-EVs was mainly about 100 nm (**Figure 1B**). To further identify the extracted extracellular vesicles, this study identified the extracellular vesicle-related proteins and ASFV proteins in ASFV-EVs. The results showed that ASFV-EVs contained extracellular vesicle-related maker proteins CD63, CD9, also had ASFV P30 and P72 proteins (**Figure 1C**). Whether ASFV DNA contained in ASFV-EVs, we further tested the ASFV protein structural protein P72 in ASFV-EVs by PCR, and the results showed that the extracellular vesicles ASFV-EVs contained the P72 nucleotide sequence (**Figure 1D**). The PCR products were verified by sequence analysis.

### ASFV-EVs Promotes the Spread of ASFV in Susceptible and Non-Susceptible Cells

**Figure 2:**
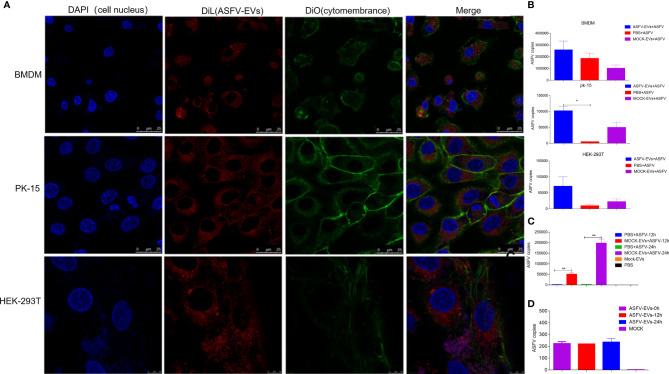
Extracellular vesicles mediate ASFV replication in ASFV-susceptible and non-susceptible cells. **(A)** Extracted ASFV-EVs was pre-stained with DiL and then inoculated into susceptible and non-susceptible cells for 8 h cell. Cell nucleus and cytomembrane were stained with DAPI (4’,6-diamidino-2-phenylindole) and DiO respectively. Meanwhile, fluorescence was measured by confocal laser microscopy. **(B)** The extracted ASFV-EVs (25ng) were inoculated into susceptible cells (BMDM) and non-susceptible cells (HEK-293T, PK-15) 8 h, meanwhile PBS was used as control, and then inoculated with ASFV(MOI:0.01). After 24 h of inoculation with ASFV, the cells and culture supernatant were collected, and DNA was extracted to detect the viral load of ASFV. **(C)** The extracted MOCK-EVs (50ng) were inoculated into PK-15 cell 8 h, meanwhile PBS was used as control, and then inoculated with ASFV(MOI:0.01), meanwhile, cells treated only with MOCK-EVs were used as negative control. At 12h and 24 h after ASFV treatment respectively, the cells and culture supernatant were collected, and DNA was extracted to detect the viral load of ASFV. **(D)** The extracted ASFV-EVs (25ng) were inoculated into BMDM cell, meanwhile PBS was used as control. At 0h 12h and 24 h after inoculated with ASFV-EVs respectively, the cells and culture supernatant were collected, and qPCR was performed to detect the p72 gene of ASFV.All data represent means ± SD, n = 3 for each group. A significant difference was calculated using a two-tailed *t*-test and labeled as *P < 0.05 and **P < 0.01 in graphs.

Studies have shown that ASFV cannot replicate in PK-15 cells. Our previous studies have found that FMDV-exosomes and SVV-exosomes mediate the spread of FMDV and SVV, respectively (Xu et al., 2020a; Xu et al., 2020b). We extracted extracellular vesicles from the plasma of ASFV-negative and ASFV-infected pigs, and the extracted extracellular vesicles (25ng) were added to susceptible (BMDM) and non-susceptible (PK-15, HEK-293T) cells before inoculation with ASFV. Confocal laser experiments performed, the results showed that extracellular vesicles transported ASFV protein into BMDM, PK-15, and HEK-293T cells (**Figure 2A**). Quantitative PCR revealed that ASFV-EVs promote the proliferation of ASFV in the susceptible and non-susceptible cells (**Figure 2B**). At the same time, we inoculated high-dose MOCK-EVs (50ng) into PK-15 cells. We found that MOCK-EVs significantly promoted the proliferation of ASFV on non-susceptibles cells 24 hours after ASFV infection (**Figure 2C**). To further explore whether ASFV-EVs contain complete ASFV virus particles, we only inoculated ASFV-EVs to MDBK cells. The results showed that the p72 gene could be detected at 0h, 12h, and 24h after being inoculated with ASFV-EVs, but the replication level of the p72 gene did not change significantly(**Figure 2D**), suggesting that ASFV-EVs did not contain complete ASFV virus particles, but incloud part of ASFV genes.

### Differentially Expressed Protein Analysis

**Figure 3:**
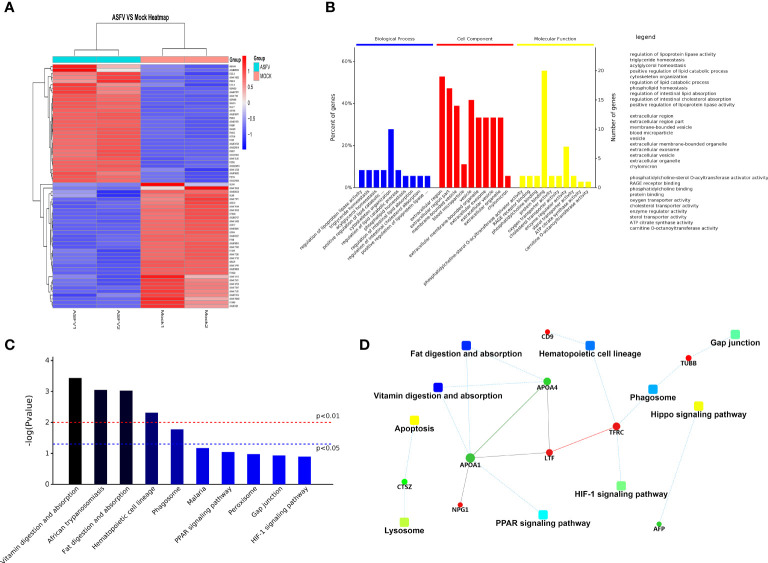
Differentially expressed protein analysis. **(A)** Differential protein cluster analysis, grouping, and categorizing data based on similarity using a clustering algorithm to classify samples and variables in two dimensions. The results show whether changes in the expression of the target proteins significantly impact the samples’ biological processing. The clustering of the target proteins distinguished proteins with different expression patterns. A subset of proteins with similar expression patterns may have a similar function, participate in the same biological pathways, or maybe be in an adjacent regulatory position in the pathways. **(B)** GO annotations for differential proteins representing Biological Process, Molecular Function, and Cellular Component. The significant enrichment analysis of GO annotations was used to evaluate the significance level of a certain GO terms enrichment by Fisher’s exact test. **(C)** Based on all qualitative proteins as the background, Fisher’s exact test was used to analyze and calculate the significance level of protein enrichment for each KEGG pathway and determine the metabolism and signaling that are significantly affected. **(D)** The dashed/solid line represents the confidence score. The default minimum value was 400, the solid line represents the larger value, the dashed line represents the smaller value, the round dot represents the protein or gene, red represents upregulation, and the green represents green down-regulation. A circle gray dot represents the comparison of more than 3 groups in which we could not obtain upregulation and down-regulation information. A giant node represents the KEGG pathway/Biological process. A yellow-blue gradient represents a significant *P*-value, yellow represents a small *P*-value, and blue represents a large *P*-value. The higher the score for each connection point, the more reliable the interaction.

Extracellular vesicles contain multiple proteins, and biologically active substances, including host cell-derived proteins and pathogen-derived proteins. We searched for host cell proteins in ASFV-EVs, and the results showed that 32 proteins were down-regulated (**Table 1**) and 34 proteins were upregulated (**Table 2**). Next, we performed a cluster analysis on the differentially expressed proteins. We clustered the proteins with similar expression patterns and similar functions (**Figure 3A**). GO analysis was performed to determine the properties of the genes and gene products using three categories: Biological Process, Molecular Function, and Cellular Component. The proteins forming the cytoskeleton organization were the most abundant for biological processes. The main biological functions are the extracellular region, membrane-bounded vesicle, blood microparticle, vesicle extracellular membrane-bounded organelle, extracellular vesicle, extracellular vesicle, extracellular organelle, and chylomicron. Protein binding and enzyme regulator activity occupied a significant cellular component (**Figure 3B**). In organisms, proteins do not perform their functions independently, but different proteins coordinate to complete a series of biochemical reactions. Therefore, pathway analysis is the most direct to develop a systematic and comprehensive understanding of cell biological processes, traits, disease mechanisms, and drug action mechanisms. KEGG is a database commonly used in genomics, and it annotates pathways based on the KEGG database for significantly differentially expressed proteins. The results showed that the main pathways involved in ASFV-EVs were vitamin digestion and absorption, African trypanosomiasis, Fat digestion and absorption, Hematopoietic cell lineage, and Phagosome malaria (**Figure 3C**). The protein interaction network analysis revealed that ASFV-EVs contained reliable interactions among the APOA4, APOA1, LTF, TFRC, and NPG1 proteins (**Figure 3D**). To further explore ASFV-derived proteins in extracellular vesicles, we searched for ASFV proteins in ASFV-EVs, and the results showed that ASFV-EVs contained 21 ASFV proteins (**Table 3**).

**Table 1:** Significantly down-regulated proteins in ASFV-EVS.

Accession	Gene Symbol	P.value
A0A481B9A6		0.003
K7GM40	APOA1	0.037
A0A4X1SG20		0.018
A0A4X1UAD2	AHSG	0.025
A0A480TGC4		0.028
A0A287BM29	APOA4	0.009
Q29014		0.016
A0SEH2		0.020
A0A480SUZ7		0.037
A0A286ZYQ7		0.001
Q8MJ76	AFP	0.014
A0A287BDV3		0.047
A0A4X1TUA7	LOC100158011	0.037
A0A4X1TZ82		0.033
F1RII6	HBE1	0.004
A0A4X1WBG8	XPO1	0.018
A0A4X1UPN1		0.001
A0A4X1T3M3	TMOD2	0.000
A0A4X1US13	GP1BB	0.008
A0A4X1TA51	CUTA	0.042
A0A4X1SMJ9	PCLO	0.043
A0A4X1TL58	KIF2B	0.001
F1RNB0	SUPT6H	0.006
F1SBY8	CROT	0.002
F1SBR0	PTPN22	0.012
I3LNV9	CLEC1B	0.011
A0A4X1V7Q2		0.002
A0A4X1T9F1	ANGPTL3	0.001
A0A287AIZ9	ABLIM1	0.000
A5GFX7	CTSZ	0.000
A0A480NSC6		0.003
I3L560	KLB	0.039
A0A4X1SP29	CMTR2	0.000
A0A4X1W194	TBC1D8	0.046

The Proteome Discoverer 2.4 software was used to calculate the TMT-labeled proteomics data quantitatively. For the significant difference analysis results, the proteins in the pairwise comparison groups that met the expression difference criteria of 1.2-fold greater with a P value less than 0.05 were regarded as significantly expressed proteins.

**Table 2:** Significantly up-regulated proteins in ASFV-EVS.

Accession	Gene Symbol	P.value
I3LQ17	PZP	0.033
A0A4X1VBD2	C4A	0.006
L8AXM9	IGHG	0.020
A0A4X1UG39	SERPINC1	0.003
F1RII7	HBB	0.015
P02067	HBB	0.037
Q8WMN8	LTF	0.039
A0A480Y8T9		0.048
P01965	HBA	0.049
K7ZPU8	IGHG5-1	0.033
A0A4X1SL55		0.004
A0A287A7G8		0.001
P62802		0.012
Q8HZV3	TFRC	0.033
P32194	NPG1	0.026
F6Q697	S100A9	0.008
P80310	S100A12	0.047
F2Z5B2	TUBB	0.011
A0A287BKF7		0.000
Q8WMQ3	CD9	0.014
A0A5G2R5E9	AZU1	0.044
F2Z5L5	HIST2H2AC	0.004
A0A480TR75		0.015
A0A5G2R318	H2AFV	0.000
A0A4X1T8I3	CAPZA1	0.025
F2Z5L6	HIST2H2AB	0.019
A0A287BED2	CEP85	0.020
Q9TR68		0.034
H9BYW4	ACLY	0.009
A0A5G2QWC4	FAM24B	0.000
A0A0B8RZ36	SPTAN1	0.037
A0A5G2RAY2	POFUT2	0.002

The Proteome Discoverer 2.4 software was used to calculate the TMT-labeled proteomics data quantitatively. For the significant difference analysis results, the proteins in the pairwise comparison groups that met the expression difference criteria of 1.2-fold greater with a P value less than 0.05 were regarded as significantly expressed proteins.

**Table 3:** ASFV protein contained in ASFV-EVs.

ID	Gene name
QGV56929.1	A137R
QGV56828.1	MGF 505-2R
QGV56940.1	MGF 110-12L
QGV56842.1	B407L
QGV56846.1	MGF 360-6L
QGV56950.1	I8L
QGV56865.1	MGF 300-4L
QGV56805.1	CP2475L
QGV56901.1	D205R
QGV56914.1	O174L
QGV56837.1	B438L
QGV56971.1	C62L
QGV56961.1	DP79L
QGV56939.1	E120R
QGV56919.1	B169L
QGV56917.1	B169L
QGV56911.1	F165R
QGV56898.1	I215L
QGV56893.1	A240L
QGV56880.1	S273R
QGV56829.1	MGF 505-6R

The protein in the plasma extracellular vesicles was compared with the ASFV protein database, and the ASFV protein contained in the extracellular vesicles was analyzed.

### Verification of Differential Proteins in Plasma Extracellular Vesicles

**Figure 4:**
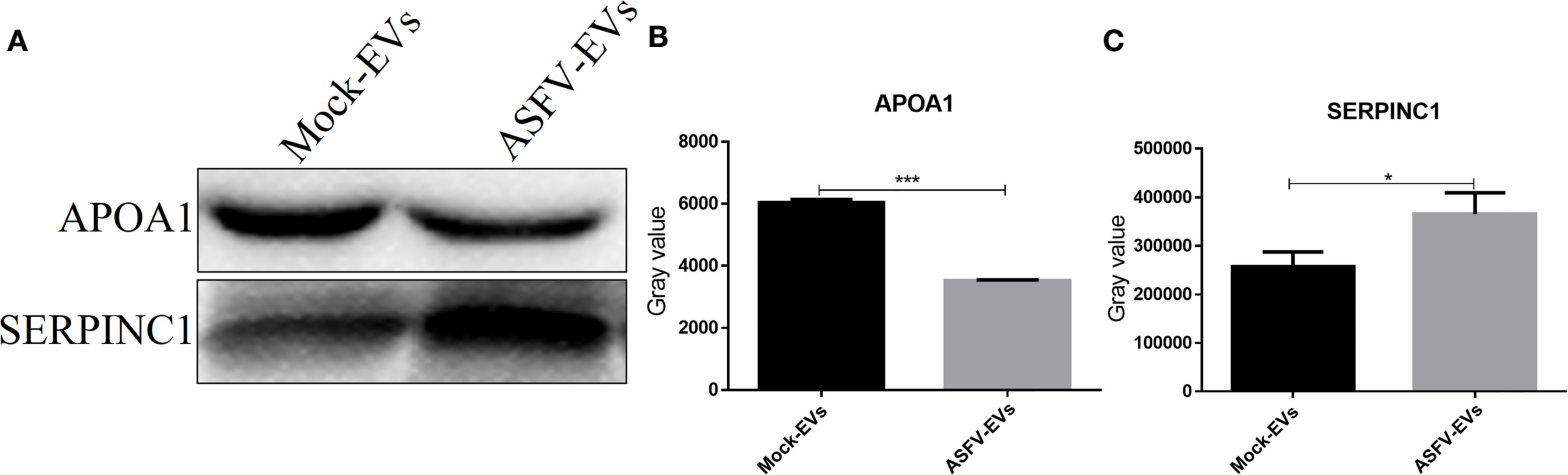
Differentially expressed protein verification. **(A)** The protein APOA1, and SERPINCI were verified by WB analysis. Before the WB experiment, the total protein concentrations of ASFV-EVs and MOCK-EVs were quantitated by measuring the UV absorbance at 280 nm, and the protein concentrations of ASFV-EVs and MOCK-EVs were adjusted to be equivalent. **(B)** The gray-scale scanning analysis of APOA1 protein in **(A)**. **(C)** The gray-scale scanning analysis of SERPINCI protein in **(A)**. The data were presented as means ± SD (n = 3 for each group). A significant difference was calculated using two-tailed t-test; *P < 0.05 and ***P < 0.001.

To further verify the differentially expressed proteins identified in extracellular vesicles, we selected APOA1 and SERPINC1 proteins in ASFV-EVs and verified by WB experiment. The results showed that both MOCK-EVs and ASFV-EVs contained the down-regulated proteins, APOA1, and the upregulated proteins, SERPINC1 (Antithrombin-III) (**Figure 4A**). Grayscale analysis of the WB results revealed that the expression APOA1 is significantly decreased (**Figure 4B**), and SERPINC1 is significantly increased in ASFV-EVs (**Figure 4C**), consistent with the results of proteomics analysis.

## Discussion

Extracellular vesicles mediate the transmission of pathogenic organisms and intercellular signals (De Paoli et al., 2018; Kalluri and LeBleu, 2020). When pathogenic microorganisms infect a host, extracellular vesicles mediate multiple signals of the pathogens as microorganisms (Cosset and Dreux, 2014; Fu et al., 2017; Claudia et al., 2018; Jan et al., 2019). Several recent studies have demonstrated that extracellular vesicles mediate the spread of pathogenic microorganisms. Porcine plasma contains many extracellular vesicles, which play an essential role in regulating cellular function (Looze et al., 2009; Lässer et al., 2011; Edward et al., 2015).

In the present study, we found for the first time that ASFV-infected pig plasma extracellular vesicles, ASFV-EVs, contained a variety of ASFV proteins, and ASFV-EVs increased the proliferation of ASFV-susceptible and non-susceptible cells. ASFV infection caused significant changes in the proteins contained within the extracellular vesicles. There are many different methods for extracting extracellular vesicles, including ultracentrifugation, gradient ultracentrifugation, and co-precipitation (Shao et al., 2018); however, ultracentrifugation is the classic method. We examined the extracted extracellular vesicles by TEM and observed a cup-shaped vesicle structure with high purity. Nanoparticle size analysis of the extracted extracellular vesicles was approximate 100 nm, indicating that the extracellular vesicles extracted in this study have higher purity, and the size consistent with literature reports (Raposo and Stoorvogel, 2013; Maas et al., 2017). There are two main types of membrane proteins, one of which is contained in almost all extracellular vesicles and may be used as a marker to distinguish extracellular vesicles from other vesicles, such as CD9, CD63, CD81. We found that ASFV-EVs contained a CD9 protein. The differentially expressed proteins in extracellular vesicles may perform critical biological functions in cells (Pisitkun et al., 2004; Raab-Traub and Dittmer, 2017; Cheruiyot et al., 2018; De Paoli et al., 2018; Jeppesen et al., 2019).

Extracellular vesicles mediate the transmission of pathogenic microorganisms. Our previous studies also found that extracellular vesicles mediate the transmission of FMDV and SVV (Zhang et al., 2019; Xu et al., 2020a; Xu et al., 2020b). Extracellular vesicles contain complete virus particles which can directly mediate viral spread. Viruses that enter cells through endocytosis can hijack and use the extracellular vesicles pathway to facilitate their survival. HCV, Zika virus, West Nile virus, and Dengue virus enter this pathway through clathrinid-mediated or receptor-mediated endocytosis (Cosset and Dreux, 2014; Alenquer and Amorim, 2015; Hamel et al., 2015; Raab-Traub and Dittmer, 2017). Several virus-infected cells secrete extracellular vesicles. Extracellular vesicles carry complete virus particles and viral genomes and proteins to help viral replication. To further study how ASFV-EVs promote the spread of ASFV, we analyzed the proteome of ASFV-EVs and showed that plasma extracellular vesicles might promote the proliferation of ASFV. Extracellular vesicles participate in signal transduction and mediate the transmission of various bioactive substances in the body (Kalluri and LeBleu, 2020), and the bioactive substances contain in extracellular vesicles from different tissues are often different. Whether the active substances contained in exosomes from different tissues can exert corresponding regulatory effects is closely related to the function of recipient cells themselves and the influence of external factors, especially after pathogenic microorganisms infect the recipient cells (Cosset and Dreux, 2014; Andrea, 2015; Harendra et al., 2015; Fu et al., 2017; Wang et al., 2017; Claudia et al., 2018; Jeppesen et al., 2019). We inoculated Mock-EVs extracted into susceptible cells BMDM cells. The results showed that Mock-EVs did not significantly promote the proliferation of ASFV. We speculated that some bioactive substances in Mock-EVs had a positive regulatory effect on the antiviral immune response of BMDM, thus affecting the replication of ASFV. However, there was no regulation or minimal regulation effect on the antiviral immunity of 293T and PK-15 cells.

Further exploration of the biologically active substances in extracellular vesicles is essential to understand the pathogenesis of ASFV. The related pathways of extracellular vesicles production and secretion determine the unique characteristics of extracellular vesicles and the complexity of their functions, especially for communication between cells. Biologically active substances in the extracellular vesicles may inhibit the immune response of host cells to promote the spread of pathogenic microorganisms. ASFV-EVs harbors a variety of ASFV proteins. Identifying differentially expressed proteins in ASFV-EVs is vital for exploring the antiviral response mechanism of host cells and the immune evasion mechanism of pathogens. Although these specific mechanisms need to be further explored, these differentially expressed proteins may be necessary for regulating the host cell processes. ASFV-EVs contain not only cell-derived proteins but also pathogen-derived proteins. We searched for ASFV proteins in ASFV-EVs and found that the extracellular vesicles wrap A137R, MGF 505-2R, MGF 110-12L, B407L, MGF 360-6L, as well as African swine fever proteins including I8L, MGF 300-4L, and CP2475L. Cellular component research is of great significance to understanding the regulation of host cells mediated by extracellular vesicles during ASFV infection. We found that the differentially expressed proteins are categorized as a biological process, molecular function, and cellular component in ASFV-EVs.

The down-regulated proteins, APOA1, KLB, TBC1D8, and the upregulated proteins, SERPINC1 (Antithrombin-III) and (H2A) H2AFV, in ASFV-EVs, were validated by WB analysis. The results showed that the above 5 proteins are present in ASFV-EVs. Unfortunately, extracellular vesicles lack an internal reference protein for WB analysis. Therefore, it is impossible to quantitate the above proteins through WB experiments. KLB is a multifunctional cytokine produced by lymphocytes and synthesized by non-lymphocytes. It plays an essential role in regulating the host immune response, the proliferation of blood cells, the defense mechanism, and the acute phase response. The expression of KLB decrease significantly in ASFV-EVs; however, its role in the antiviral immune response needs to be further verified. Our previous studies found that FMDV could eventually inhibit the secretion of host cell extracellular vesicles by degrading the RAB27A protein. Extracellular vesicles could regulate the host cell immune response mediated by biologically active substances and inhibit pathogenic microorganisms’ proliferation through miRNA. TBC family proteins are in the cytoplasm and are mainly involved in endocytosis or vesicle transport. Overexpression of TBCW8B results in enhanced cell proliferation, migration, and infiltration, suggesting that after ASFV infection of host cells, it regulate TBCW8B to inhibit the secretion of host cell extracellular vesicles.

## Conclusion

In this study, extracellular vesicles were extracted and identified from ASFV-positive pig plasma for the first time. We found that the extracted extracellular vesicles contained the ASFV genome and proteins. Proteomic analysis of ASFV-EVs revealed that ASFV causes significant changes in extracellular vesicles-containing proteins contained. ASFV-EVs promote the spread of ASFV in susceptible and non-susceptible cells. This study provided novel information for the further study of ASFV infection and immune regulation *in vivo*.

## Data Availability Statement

The original contributions presented in the study are included in the article/**Supplementary Material**. Further inquiries can be directed to the corresponding author.

## Ethics Statement

The animal study was reviewed and approved by Animal Ethics Committee of Lanzhou Veterinary Research Institute of the Chinese Academy of Agricultural Sciences. Written informed consent was obtained from the owners for the participation of their animals in this study.

## Author Contributions

GX and KZ carried out most the experiments and wrote the manuscript. CS, BY, HL, TZ, XS, JY, DZ, HC, XY, XC and YH participated in the clinical evaluation and performed the calculations and estimations. HZ, XL, and KZ conceived the study, participated in its design and coordination, and revised the manuscript. All the authors read and approved the final manuscript.

## Conflict of Interest

The authors declare that the research was conducted in the absence of any commercial or financial relationships that could be construed as a potential conflict of interest.

## Publisher’s Note

All claims expressed in this article are solely those of the authors and do not necessarily represent those of their affiliated organizations, or those of the publisher, the editors and the reviewers. Any product that may be evaluated in this article, or claim that may be made by its manufacturer, is not guaranteed or endorsed by the publisher.

## Supplementary Material

Supplementary Data Attachment-ASFV-EVs Protein Quantitative and Significant Difference Analysis Table.
